# Rice-Map: a new-generation rice genome browser

**DOI:** 10.1186/1471-2164-12-165

**Published:** 2011-03-30

**Authors:** Jun Wang, Lei Kong, Shuqi Zhao, He Zhang, Liang Tang, Zhe Li, Xiaocheng Gu, Jingchu Luo, Ge Gao

**Affiliations:** 1 Center for Bioinformatics, National Laboratory of Protein Engineering and Plant Genetic Engineering, College of Life Sciences, Peking University, Beijing, 100871, PR China; 2 STG Lab Based Services, China Development Lab, IBM (China) Investment Co Limited, Beijing 100193, PR China; 3 Laboratory of Systematic and Evolutionary Botany, Institute of Botany, Chinese Academy of Sciences, Beijing 100093, PR China

## Abstract

**Background:**

The concurrent release of rice genome sequences for two subspecies (*Oryza sativa *L. ssp. *japonica *and *Oryza sativa *L. ssp. *indica*) facilitates rice studies at the whole genome level. Since the advent of high-throughput analysis, huge amounts of functional genomics data have been delivered rapidly, making an integrated online genome browser indispensable for scientists to visualize and analyze these data. Based on next-generation web technologies and high-throughput experimental data, we have developed Rice-Map, a novel genome browser for researchers to navigate, analyze and annotate rice genome interactively.

**Description:**

More than one hundred annotation tracks (81 for *japonica *and 82 for *indica*) have been compiled and loaded into Rice-Map. These pre-computed annotations cover gene models, transcript evidences, expression profiling, epigenetic modifications, inter-species and intra-species homologies, genetic markers and other genomic features. In addition to these pre-computed tracks, registered users can interactively add comments and research notes to Rice-Map as User-Defined Annotation entries. By smoothly scrolling, dragging and zooming, users can browse various genomic features simultaneously at multiple scales. On-the-fly analysis for selected entries could be performed through dedicated bioinformatic analysis platforms such as WebLab and Galaxy. Furthermore, a BioMart-powered data warehouse "Rice Mart" is offered for advanced users to fetch bulk datasets based on complex criteria.

**Conclusions:**

Rice-Map delivers abundant up-to-date *japonica *and *indica *annotations, providing a valuable resource for both computational and bench biologists. Rice-Map is publicly accessible at http://www.ricemap.org/, with all data available for free downloading.

## Background

Rice (*Oryza sativa*) is one of the most important grain crops, being staple food for massive people, especially those in Asian, Latin American, and African countries [1]. In addition to its economical importance, rice is an ideal model organism for studies on other cereal crops like sorghum (*Sorghum bicolor*), wheat (*Triticum aestivum*) and maize (*Zea mays*) [2,3] because of its relative small genome size, abundant sequences available in public databases, well studied genetic markers and homologous relationship with other cereal crops.

The release of two draft sequences of the rice genome in 2002 effectively boosts research on rice biology [4,5]. The whole genome sequences for two cultivated subspecies *Oryza sativa *L. ssp. *japonica *[4] and *Oryza sativa *L. ssp. *indica *[5] enable scientists to systematically investigate the molecular basis of rice biology at the whole genome level [6]. They also allow researchers to unravel genetic basis for phenotypic differences between *japonica *and *indica*, such as the protein and amylose equivalence of seeds, the grain yield and nitrogen utilization [7,8]. Importantly, the short divergence time between the two subspecies offers great opportunities for comparative genomic studies [6,9]. In addition to genome data, the advent of high-throughput profiling technology makes it possible to deliver large-scale transcriptome and epigenomic profiling data rapidly and cost-effectively [6,10,11]. These data provide a snapshot for the transcriptome activity in a range of tissues and developmental stages, revealing the dynamics of rice genome widely [12].

The availability of rice genomes provides a natural framework to organize and access various annotations generated by functional and genetic analysis [6,11]. Since the release of rice genomes [4,5], several rice genome browsers have been constructed and made public to the rice research community. The Rice Genome Annotation Project at Michigan State University (MSU) has developed a genome browser based on GBrowse system [13], integrating a curated gene set and more than seventy annotation tracks [14,15]. Although SNPs between *japonica *and *indica *genomes have been presented, no annotation for *indica *genome is available in the current MSU genome browser. Similarly, RAP-DB offers many annotations for *japonica *genome [16], while *indica *annotation is not presented. Being designed to be a plant comparative genomic platform, Gramene has integrated abundant genetic and functional annotations for the two sequenced cultivated rice and their wild relatives [17]. By employing the well-established Ensembl software system, Gramene provides rich user experience with close integration to other Ensembl-based portals like the newly-released Ensembl Genomes [18]. However, there are only limited transcriptome and epigenetic annotations available in the existing rice genome browsers, especially for the *indica *genome.

Thus, we have developed Rice-Map, a novel rice genome browser. Currently, Rice-Map has integrated more than one hundred annotation tracks for *japonica *and *indica*. These tracks cover gene models, transcript evidences, expression profiling, epigenetic modification markers, inter-species and intra-species homologies, genetic markers and other genomic features, providing a valuable resource for both computational and bench biologists. Besides these pre-computed tracks, user-supplied comments and annotations can be added to Rice-Map instantly. Built with next-generation web technologies, Rice-Map allows biologists to navigate rice genome annotations in a highly-interactive approach. In addition to browsing, annotation entries could be sent to dedicated bioinformatic analysis platforms for further analysis. Advanced users can fetch bulk datasets through a BioMart-powered [19] data warehouse "Rice Mart".

## Construction and Content

**Figure 1:**
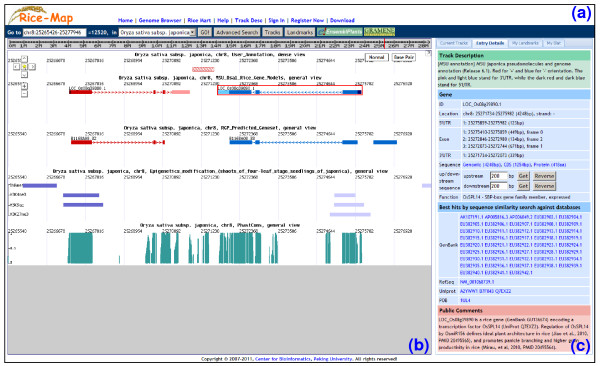
**Main interface of the Rice-Map genome browser**. (a) Rice-Map location toolbar which consists of various buttons for users to control the behaviors of Rice-Map. (b) Centric map panel. By smoothly scrolling, dragging and zooming, users can browse various genomic features at multiple scales in graphic or basepair view, fine-tuning can be achieved by using buttons in the navigation control panel at the upper left corner. (c) Information panel, including several tabs for displaying entry details and other information.

Similar to other popular genome browsers [13,20,21], Rice-Map presents various pre-computed annotations as tracks (Figure 1). Currently, 81 tracks for *japonica *and 82 tracks for *indica *are available in Rice-Map, including predicted rice gene models, transcriptome data, inter-species and intra-species homologies, genetic markers and other genomic features like repeat elements (Supplementary Figure S1 in Additional File 1). Besides 24 tracks directly imported from public data resources, 139 annotation tracks have been computed locally. All detailed annotation methodologies are available at http://www.ricemap.org/tracks/.

### Gene Annotation

One of the most challenging issues in genome annotation is to identify a comprehensive gene set encoded by the genome [2,14]. Rice-Map currently has integrated various well-known gene model annotations generated by MSU [3], RGP (Rice Genome Project) [22] and BGI (Beijing Genomics Institute) [23]. BLAT [24] has been employed to map these gene models to chromosomes, unless the coordinates are provided by the original source. Similar to previous studies [16,25], inconsistencies among different gene sets have been found (Supplementary Figure S2 in Additional File 1), suggesting the value of presenting multiple gene annotations simultaneously. In addition to protein-coding genes, recent studies have revealed that non-coding RNAs (ncRNAs) play key roles in various plant physiological and developmental processes [26,27]. Totally, 114,048 non-coding RNAs have been downloaded from NONCODE[28], miRBase [29] and CSRDB [30]. After removing redundancies, 100,485 and 72,035 of non-coding RNAs have been mapped to the *japonica *and *indica *genome, respectively.

### Transcriptome Annotation

Full-length cDNA and expressed sequence tag (EST) sequences provide direct transcriptional evidence for predicted genes, and allow deduction of their alternative splicing patterns [25]. We have downloaded 39,531 cDNAs (30,436 for *japonica *and 9,095 for *indica*) and 1,186,900 ESTs (985,283 for *japonica *and 201,617 for *indica*) from GenBank, and mapped them to respective genomes with BLAT [24]. More than 88% cDNAs and 60% ESTs have been mapped uniquely under the criteria of identity > 96%, coverage > 90% and score >= 30. For the remaining dataset, 1,632 *japonica *and 455 *indica *cDNAs, as well as 47,549 *japonica *and 12,934 *indica *ESTs have been mapped to multiple locations, implying the results of genome duplication events or the relics of pseudogenes [31]. We have taken a conservative approach to filter these ambiguous hits based on the alignment quality, keeping only the one(s) with highest quality score [32]. Finally, 38,699 cDNAs (30,261 for *japonica *and 8,438 for *indica*) and 1,024,764 ESTs (891,194 for *japonica *and 133,570 for *indica*) have been mapped, covering 42.31% of the *japonica *and 22.41% of the *indica *genome, respectively.

The expression data provide a snapshot for the transcriptome activity in various tissues and developmental stages, enabling researchers to understand the complex dynamics of rice genome, such as expression patterns, function regulation and the potential transcribed regions [33]. Currently, expression data of 4 tissues for *japonica *and 15 tissues for *indica *have been downloaded from the GEO microarray database [34] and integrated into our browser, covering Anther development, Pollination Fertilization, Early embryogenesis, Germinating seed, Endosperm, Seedling (Plumule and Radicle) and Shoot (Vegetative tissues, Callus, Stem, Leaf, Sheath and Panicle). Furthermore, mRNA-Seq expression data of four-leaf stage seedling shoots provided by global epigenetic and transcriptional experiment analysis [12] have also been imported into Rice-Map. These mRNA-Seq data were mapped to the genome using tophat [35], and assembled into transcripts by Cufflinks [36]. Totally, the mapped expression data cover 78.82% and 87.16% of annotated *japonica *and *indica *gene models, providing further support for 98.12% and 90.29% of the *japonica *and *indica *cDNAs. In addition, the integrated mRNA-Seq data also identified 3,723 intergenic Transcriptionally Active Regions (iTARs) in *japonica *and 9,762 iTARs in *indica*, providing a valuable resource for further gene hunting and functional screening.

Epigenetic modifications are essential for complex regulatory machinery of differential gene expression [12]. DNA methylation in transcribed regions is regarded essential for epigenetic regulation, maintaining genomic stability [12]. Histone modification plays an important role in gene expression regulation by changing chromatin status and recruiting transcription related protein complexes [12]. Rice-Map have integrated epigenetic modification annotations derived from high-throughput profiling data in shoots of four-leaf stage seedlings for both *japonica *and *indica *genomes, including both DNA methylation and histone modification data (H3K4me3, H3K9ac and H3K27me3) [12]. Finally, 34,378 and 42,152 DNA methylation modification regions for *japonica *and *indica *were called using MACS [37], covering 14.39% of the *japonica *genome and 11.80% of the *indica *genome. For the histone modification, 88,474 and 89,243 regions were detected, covering 13.36% and 12.95% of the *japonica *and *indica *genome, respectively. Being the first integration of these valuable data with other transcriptome annotation, Rice-Map provides a genome-wide profiling vision for the complex rice transcriptome.

### Comparative Genomics Annotation

Cross-species comparison offers additional insights into rice biology [10]. A total of 225,224 assembled PlantGDB transcripts [38] from various plant species including *Arabidopsis thaliana*, *Sorghum bicolor *and *Brachypodium distachyon *have been mapped to the rice genome using GMAP [39], covering 26.05% of the *japonica *and 26.95% of the *indica *genome, respectively. Meanwhile, 18.71% and 15.41% (151,139 and 124,439 proteins out of 807,731) UniProt plant proteins have been aligned to the *japonica *and *indica *genomes respectively, providing clues for identifying novel genes. Besides homologs to known sequences, base-level conservation is another indicator for functional important regions [40,41]. Pair-wise conservative scores between *japonica *and nine representative plants (*Arabidopsis thaliana*, *Brachypodium distachyon*, *Cucumis sativus*, *Mimulus guttatus*, *Zea mays*, *Populus trichocarpa*, *Sorghum bicolor*, *Glycine max *and *Vitis vinifera*) derived from VISTA pair-wise genome alignments [42] have also been integrated into Rice-Map. Moreover, we have further constructed multiple genome alignments across three sequenced grass genomes (rice, *Sorghum bicolor *and *Brachypodium distachyon*) and inferred PhastCons scores, accordingly [43].

The genome-wide comparison between *japonica *and *indica *offers valuable clues for rice improvement [6,11]. By constructing pair-wise chromosome alignment between *japonica *and *indica *genome sequences, we have screened more than 1.4 million SNPs between *japonica *and *indica *using the NUCMER SNP pipeline [44], nearly four times the number previously reported [9]. We have further identified large colinear blocks among the two subspecies (1,137 in *japonica *and 1,112 in *indica*), presenting a global view of the chromosomal evolution since their divergence [45].

### Genetic Marker Annotation

Genetic markers lay the foundation for genetic mapping and marker-assisted selection of agriculturally important traits [46]. High density genetic markers are crucial for fine mapping of causal variation that may contribute to quality improvement in rice cultivars and crop breeding [7,47]. To facilitate selecting potential polymorphic markers, Rice-Map integrates four genetic marker tracks, with an average of 11.39 markers for *japonica *and 11.05 markers for *indica *per 100 kb nucleotides, respectively. In addition to these genetic markers, Rice-Map also integrates rice QTL data generated by the Gramene QTL database [17,48], providing direct connections between genetic markers and traits [49].

### User-Defined Annotation (UDA)

It is mostly impractical to import all biological annotations into the core Rice-Map database. In addition to integrating the pre-computed annotation tracks, Rice-Map provides a User-Defined Annotation mechanism for users to add their own annotations. Registered users can quickly add their own notes to Rice-Map, and choose to make them private or public.

Firstly, registered users can write comments for existing entries (Supplementary Figure S3a in Additional File 1). Besides plain text, users can format their comments in various fonts/colors, organize multiple items as list and add external links in a Microsoft Word like editor. Advanced users can also input HTML tags online for more sophisticated layout. Users are encouraged to contribute new valuable annotation for any genomic region to Rice-Map via the "User Annotation" track (Supplementary Figure S3b in Additional File 1). By selecting interesting regions with the "magic wand" tool (see below), registered users can add new annotation entries interactively. All these user-defined annotation entries will be displayed in the "User Annotation" track, which could be manipulated exactly in the same way as the pre-computed tracks. Furthermore, by adding stars and writing reviews, users can evaluate the quality and importance of a track, providing a community-based feedback mechanism (Supplementary Figure S3c in Additional File 1).

Users have complete control over their comments and annotations by setting them for public access or for private only. While public entries can be viewed by all users, private entries can be only viewed by the owner as personal research notes. For user convenience, a web interface is provided for exporting the publicly available user comments and annotations.

## Utility and Discussion

### Navigate the genome

Based on next-generation web technologies, Rice-Map allows users to navigate the whole genome interactively through a Google maps like interface. By smoothly scrolling, dragging and zooming, users can browse various genomic features at multiple scales.

The web interface of Rice-Map is designed to be like Google maps (Figure 1). Besides jumping directly to a specified chromosome location, users can also search a gene through its ID/function, or locate inputted sequences by BLAT [24] using the "Advanced Search" dialog box. By clicking the "Tracks" button, tracks can be switched on/off for display in the centric map panel.

Users can move around by dragging the map directly, and fine-tuning with the arrow buttons in the navigation control panel (at the upper left corner). By clicking the two buttons with plus sign ("+") or minus sign ("-"), users can zoom in or out without reloading the whole page. Clicking the "Base pair" button at the upper right corner enables a special view in single-base resolution. And users with small screen can use full screen view mode for larger view area by clicking the upper right corner arrow.

Clicking an annotation entry shows its detailed information in the "Entry Details" tab of the right information panel. The exact content displayed for a given entry depends on the available annotation. For most entries, it includes entry ID, location, CDS/protein/genomic sequence and public comments contributed by other users.

**Figure 2:**
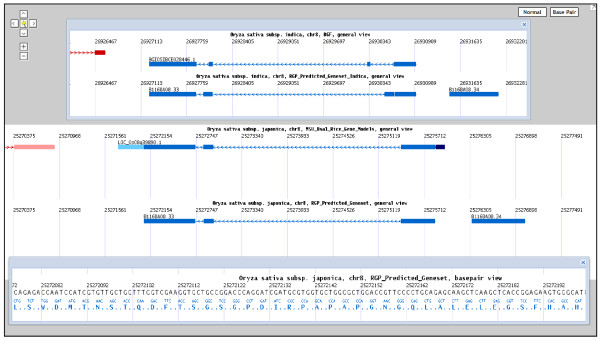
**Split-window view of multiple regions for comparative analysis**. Rice-Map supports split-window view of multiple regions to facilitate comparative analysis among different genomic regions. User can select an interesting chromosome region with the "magic wand" tool and view the region in a new sub-window. For example, user can view the *OsSPL14 *gene (LOC_Os08g39890.1) in *japonica *and find its best gene hit in *indica *(BGIOSIBCE028446.1) by the hint of "Best Indica Hit" track. Then user can view these two genes simultaneously in different windows and arrange them freely. The region can also be displayed in basepair view in another sub-window to present the detailed nucleotide information beneath the graphic annotation.

With the multiple-functional "magic wand" tool, users can select and inspect interesting regions interactively. After clicking the centric "magic wand" icon at navigation control panel and selecting an interesting region by mouse, available operations will be listed in a pop-up menu. Besides in-place zooming in the centric map panel, selected region can also be displayed in a new sub-window. The sub-window is operated independently to the main centric map panel, facilitating comparison between different chromosomal areas (Figure 2). In addition, the menu also provides option for registered users to write User-Defined Annotation for the selected region.

### Analyze the genome

Rice-Map provides various ways to access annotation data other than the browser interface described above. For researchers working with large volumes of data, Rice-Map provides a BioMart-powered data warehouse [19,50] called "Rice Mart" for fetching bulk data based on complex criteria (Supplementary Figure S4 in Additional File 1). Skillful bioinformaticians can write scripts to fetch data through standard Mart-API [50]. And all pre-computed tracks can be downloaded as tab delimited text files at http://www.ricemap.org/download/.

Bioinformatic analysis is often needed after getting desired data. Rather than integrating comprehensive bioinformatic analysis tools, Rice-Map allows users to perform analysis on specified annotations data by launching dedicated bioinformatic analysis platforms like WebLab [51] or Galaxy [52]. Entry-related nucleotide or protein sequences could be sent out for analysis by clicking link in the "Entry Details" tab (Supplementary Figure S5a in Additional File 1). Even more flexibly, users can select interesting genomic regions interactively by "magic wand" tool and submit the selected genomic sequence to external bioinformatic platforms. For the convenience of researchers dealing with bulk data, the results of batch query in Rice Mart can also be sent to external bioinformatic platforms (Supplementary Figure S5b in Additional File 1).

It is not unusual to become "lost in the map" after a series of dragging, searching and opening/closing tracks. Rice-Map allows users to create "landmark" to record current location and track configuration. Users can return to previous browsing status at any time by clicking the landmark name listed in the "My landmarks" tab of the right information panel. A similar feature is provided for navigating among multiple BLAT search results in the "My Blat" tab of the right information panel.

### The value of *indica *annotation

The release of *japonica *and *indica *genome drafts [4,5], as well as the rapidly delivered high-throughput data effectively promotes research on rice biology. Since *japonica *and *indica *are highly related but biologically distinct subspecies, detailed annotations for *indica *are as important as those for *japonica *[53]. Comparative study for these two subspecies offers unique opportunity for both biological and agricultural research, such as the phenotypic differences between these two subspecies [8], rice domestication research [54] and the improvement of rice yield [11]. Recently, an ideal rice architecture gene *OsSPL14 *[55] and another gene which increased yield during rice domestication [56] have been identified from QTL analysis derived from cross/backcross between *japonica *and *indica *lines. These researches clearly demonstrate the value of *indica *resource. On the other hand, while extensive *japonica *annotations have been presented in several widely used genome browsers [14,16,17], only limited *indica *annotations are available publicly [17,23], hindering comparative research. Rice-Map provides novel data to the research community by integrating not only *japonica *but also *indica *annotation into a uniform highly-interactive interface, facilitating comparative genomic studies of these two subspecies. By integrating comprehensive rice genome data for both sequenced subspecies, Rice-Map constitutes a valuable online resource for the rice community.

## Conclusions

Built with next-generation web technologies and high-throughput experimental data, Rice-Map provides a highly-interactive user interface for researchers to navigate, analyze and annotate the rice genome. Currently, Rice-Map has integrated more than one hundred annotation tracks for *japonica *and *indica*, providing a valuable resource for both computational and bench biologists. By embracing high-throughput functional genomics data for both *japonica *and *indica *genomes, Rice-Map effectively enables researchers to investigate the dynamics of the rice genome. Aiming to be a comprehensive rice genome annotation resource, Rice-Map is constantly incorporating new data and up-to-date annotations with the growth of our knowledge. Regular updating to the backend database is scheduled four times per year, with new annotation branch forked as long as new genome assembly comes out. Rice-Map source codes are publicly available at the download page http://www.ricemap.org/download/ under the GNU General Public License v3.0, and we will continue to improve the underlying architecture for better visualization and usability.

## Availability and Requirements

Rice-Map is an open rice genome browser publicly accessible at http://www.ricemap.org/, with all the pre-computed annotation data freely downloadable for further computational analysis. Based on our test, Rice-Map is compatible with the most common web browsers such as Mozilla Firefox (version 3), Internet Explorer (version 7, 8), Apple Safari (version 5) and Google Chrome (version 6, 7, 8, 9).

## Authors' contributions

JW and LK conceived the research and drafted the paper, SQZ, HZ, LT, ZL and XCG conceived the research and helped to revise the manuscript; GG and JCL conducted the research and wrote the article. All authors have read and approved the final manuscript.

## Supplementary Material

Additional file 1 **Rice-Map supplementary figures**. Supplementary figures about number of pre-computed tracks, inconsistent areas among different gene annotation tracks and Rice-Map user operations. Click here for file

## References

[B1] Kennedy D The importance of rice Science 2002 296 5565 13 10.1126/science.296.5565.13 11934991

[B2] International Rice Genome Sequencing Project The map-based sequence of the rice genome Nature 2005 436 7052 793 800 10.1038/nature03895 16100779

[B3] Yuan Q Ouyang S Wang A Zhu W Maiti R Lin H Hamilton J Haas B Sultana R Cheung F The institute for genomic research Osa1 rice genome annotation database Plant Physiol 2005 138 1 18 26 10.1104/pp.104.059063 15888674 PMC1104156

[B4] Goff SA Ricke D Lan TH Presting G Wang R Dunn M Glazebrook J Sessions A Oeller P Varma H A draft sequence of the rice genome (Oryza sativa L. ssp. japonica) Science 2002 296 5565 92 100 10.1126/science.1068275 11935018

[B5] Yu J Hu S Wang J Wong GK Li S Liu B Deng Y Dai L Zhou Y Zhang X A draft sequence of the rice genome (Oryza sativa L. ssp. indica) Science 2002 296 5565 79 92 10.1126/science.1068037 11935017

[B6] Jung KH An G Ronald PC Towards a better bowl of rice: assigning function to tens of thousands of rice genes Nat Rev Genet 2008 9 2 91 101 18160965 10.1038/nrg2286

[B7] Singh V Okadome H Toyoshima H Isobe S Ohtsubo K Thermal and physicochemical properties of rice grain, flour and starch J Agric Food Chem 2000 48 7 2639 2647 10.1021/jf990374f 10898601

[B8] Ntanos DA K SD Genotypic differences for grain yield and nitrogen utilization in Indica and Japonica rice under Mediterranean conditions Field Crops Research 2002 83 83 251 260

[B9] Feltus FA Wan J Schulze SR Estill JC Jiang N Paterson AH An SNP resource for rice genetics and breeding based on subspecies indica and japonica genome alignments Genome Res 2004 14 9 1812 1819 10.1101/gr.2479404 15342564 PMC515328

[B10] Hirano H-Y Hirai A (eds) Rice Biology in the Genomics Era 2008 Springer

[B11] Xing Y Zhang Q Genetic and molecular bases of rice yield Annu Rev Plant Biol 2010 61 421 442 10.1146/annurev-arplant-042809-112209 20192739

[B12] He G Zhu X Elling AA Chen L Wang X Guo L Liang M He H Zhang H Chen F Global epigenetic and transcriptional trends among two rice subspecies and their reciprocal hybrids Plant Cell 22 1 17 33 10.1105/tpc.109.072041 20086188 PMC2828707

[B13] Stein LD Mungall C Shu S Caudy M Mangone M Day A Nickerson E Stajich JE Harris TW Arva A The generic genome browser: a building block for a model organism system database Genome Res 2002 12 10 1599 1610 10.1101/gr.403602 12368253 PMC187535

[B14] Ouyang S Zhu W Hamilton J Lin H Campbell M Childs K Thibaud-Nissen F Malek RL Lee Y Zheng L The TIGR Rice Genome Annotation Resource: improvements and new features Nucleic Acids Res 2007 35 Database D883 887 10.1093/nar/gkl976 17145706 PMC1751532

[B15] Yuan Q Ouyang S Liu J Suh B Cheung F Sultana R Lee D Quackenbush J Buell CR The TIGR rice genome annotation resource: annotating the rice genome and creating resources for plant biologists Nucleic Acids Res 2003 31 1 229 233 10.1093/nar/gkg059 12519988 PMC165506

[B16] Tanaka T Antonio BA Kikuchi S Matsumoto T Nagamura Y Numa H Sakai H Wu J Itoh T Sasaki T The Rice Annotation Project Database (RAP-DB): 2008 update Nucleic Acids Res 2008 36 Database D1028 1033 18089549 10.1093/nar/gkm978 PMC2238920

[B17] Liang C Jaiswal P Hebbard C Avraham S Buckler ES Casstevens T Hurwitz B McCouch S Ni J Pujar A Gramene: a growing plant comparative genomics resource Nucleic Acids Res 2008 36 Database D947 953 17984077 10.1093/nar/gkm968 PMC2238951

[B18] Kersey PJ Lawson D Birney E Derwent PS Haimel M Herrero J Keenan S Kerhornou A Koscielny G Kahari A Ensembl Genomes: extending Ensembl across the taxonomic space Nucleic Acids Res 2010 38 Database D563 569 10.1093/nar/gkp871 19884133 PMC2808935

[B19] Haider S Ballester B Smedley D Zhang J Rice P Kasprzyk A BioMart Central Portal--unified access to biological data Nucleic Acids Res 2009 37 Web Server W23 27 10.1093/nar/gkp265 19420058 PMC2703988

[B20] Birney E Ensembl: a genome infrastructure Cold Spring Harb Symp Quant Biol 2003 68 213 215 10.1101/sqb.2003.68.213 15338620

[B21] Karolchik D Baertsch R Diekhans M Furey TS Hinrichs A Lu YT Roskin KM Schwartz M Sugnet CW Thomas DJ The UCSC Genome Browser Database Nucleic Acids Res 2003 31 1 51 54 10.1093/nar/gkg129 12519945 PMC165576

[B22] Namiki N Antonio BA Idonuma A Masukawa MShibata M Ito Y Yamamoto M Ohta I Mukai Y Naitoh S Katayose Y Matsumoto T Nagamura Y Sasaki T Rice Genome Annotation Pipeline System in RGP Rice Genetics Newsletter 2003 20 119 121

[B23] Zhao W Wang J He X Huang X Jiao Y Dai M Wei S Fu J Chen Y Ren X BGI-RIS: an integrated information resource and comparative analysis workbench for rice genomics Nucleic Acids Res 2004 32 Database D377 382 10.1093/nar/gkh085 14681438 PMC308819

[B24] Kent WJ BLAT--the BLAST-like alignment tool Genome Res 2002 12 4 656 664 11932250 10.1101/gr.229202 PMC187518

[B25] Liang C Mao L Ware D Stein L Evidence-based gene predictions in plant genomes Genome Res 2009 19 10 1912 1923 10.1101/gr.088997.108 19541913 PMC2765265

[B26] Brosnan CA Voinnet O The long and the short of noncoding RNAs Curr Opin Cell Biol 2009 21 3 416 425 10.1016/j.ceb.2009.04.001 19447594

[B27] Bonnet E Wuyts J Rouze P Van de Peer Y Detection of 91 potential conserved plant microRNAs in Arabidopsis thaliana and Oryza sativa identifies important target genes Proc Natl Acad Sci USA 2004 101 31 11511 11516 10.1073/pnas.0404025101 15272084 PMC509231

[B28] Liu C Bai B Skogerbo G Cai L Deng W Zhang Y Bu D Zhao Y Chen R NONCODE: an integrated knowledge database of non-coding RNAs Nucleic Acids Res 2005 33 Database D112 115 15608158 10.1093/nar/gki041 PMC539995

[B29] Griffiths-Jones S Saini HK van Dongen S Enright AJ miRBase: tools for microRNA genomics Nucleic Acids Res 2008 36 Database D154 158 17991681 10.1093/nar/gkm952 PMC2238936

[B30] Johnson C Bowman L Adai AT Vance V Sundaresan V CSRDB: a small RNA integrated database and browser resource for cereals Nucleic Acids Res 2007 35 Database D829 833 10.1093/nar/gkl991 17169981 PMC1781248

[B31] Wang X Shi X Hao B Ge S Luo J Duplication and DNA segmental loss in the rice genome: implications for diploidization New Phytol 2005 165 3 937 946 10.1111/j.1469-8137.2004.01293.x 15720704

[B32] Kent WJ Sugnet CW Furey TS Roskin KM Pringle TH Zahler AM Haussler D The human genome browser at UCSC Genome Res 2002 12 6 996 1006 12045153 10.1101/gr.229102 PMC186604

[B33] Lu T Lu G Fan D Zhu C Li W Zhao Q Feng Q Zhao Y Guo Y Huang X Function annotation of the rice transcriptome at single-nucleotide resolution by RNA-seq Genome Res 20 9 1238 1249 10.1101/gr.106120.110 20627892 PMC2928502

[B34] Barrett T Troup DB Wilhite SE Ledoux P Evangelista C Kim IF Tomashevsky M Marshall KA Phillippy KH Sherman PM NCBI GEO: archive for functional genomics data sets--10 years on Nucleic Acids Res10.1093/nar/gkq1184 PMC3013736 21097893

[B35] Trapnell C Pachter L Salzberg SL TopHat: discovering splice junctions with RNA-Seq Bioinformatics 2009 25 9 1105 1111 10.1093/bioinformatics/btp120 19289445 PMC2672628

[B36] Trapnell C Williams BA Pertea G Mortazavi A Kwan G van Baren MJ Salzberg SL Wold BJ Pachter L Transcript assembly and quantification by RNA-Seq reveals unannotated transcripts and isoform switching during cell differentiation Nat Biotechnol 28 5 511 515 10.1038/nbt.1621 20436464 PMC3146043

[B37] Zhang Y Liu T Meyer CA Eeckhoute J Johnson DS Bernstein BE Nusbaum C Myers RM Brown M Li W Model-based analysis of ChIP-Seq (MACS) Genome Biol 2008 9 9 R137 10.1186/gb-2008-9-9-r137 18798982 PMC2592715

[B38] Duvick J Fu A Muppirala U Sabharwal M Wilkerson MD Lawrence CJ Lushbough C Brendel V PlantGDB: a resource for comparative plant genomics Nucleic Acids Res 2008 36 Database D959 965 18063570 10.1093/nar/gkm1041 PMC2238959

[B39] Wu TD Watanabe CK GMAP: a genomic mapping and alignment program for mRNA and EST sequences Bioinformatics 2005 21 9 1859 1875 10.1093/bioinformatics/bti310 15728110

[B40] Bejerano G Pheasant M Makunin I Stephen S Kent WJ Mattick JS Haussler D Ultraconserved elements in the human genome Science 2004 304 5675 1321 1325 10.1126/science.1098119 15131266

[B41] Kellis M Patterson N Endrizzi M Birren B Lander ES Sequencing and comparison of yeast species to identify genes and regulatory elements Nature 2003 423 6937 241 254 10.1038/nature01644 12748633

[B42] Frazer KA Pachter L Poliakov A Rubin EM Dubchak I VISTA: computational tools for comparative genomics Nucleic Acids Res 2004 32 Web Server W273 279 10.1093/nar/gkh458 15215394 PMC441596

[B43] Siepel A Bejerano G Pedersen JS Hinrichs AS Hou M Rosenbloom K Clawson H Spieth J Hillier LW Richards S Evolutionarily conserved elements in vertebrate, insect, worm, and yeast genomes Genome Res 2005 15 8 1034 1050 10.1101/gr.3715005 16024819 PMC1182216

[B44] Kurtz S Phillippy A Delcher AL Smoot M Shumway M Antonescu C Salzberg SL Versatile and open software for comparing large genomes Genome Biol 2004 5 2 R12 10.1186/gb-2004-5-2-r12 14759262 PMC395750

[B45] Wang X Shi X Li Z Zhu Q Kong L Tang W Ge S Luo J Statistical inference of chromosomal homology based on gene colinearity and applications to Arabidopsis and rice BMC Bioinformatics 2006 7 447 10.1186/1471-2105-7-447 17038171 PMC1626491

[B46] Hubbard T Barker D Birney E Cameron G Chen Y Clark L Cox T Cuff J Curwen V Down T The Ensembl genome database project Nucleic Acids Res 2002 30 1 38 41 10.1093/nar/30.1.38 11752248 PMC99161

[B47] Keiko Hatae SA Midori Kasai Textural differences between indica and japonica varieties in cooked rice Rice is life: scientific perspectives for the 21st century 2005 253 256

[B48] Ni J Pujar A Youens-Clark K Yap I Jaiswal P Tecle I Tung CW Ren L Spooner W Wei X Gramene QTL database: development, content and applications Database (Oxford) 2009 2009 bap005 20157478 10.1093/database/bap005 PMC2790302

[B49] Ntanos SDKaDA Genotypic differences for grain yield and nitrogen utilization in Indica and Japonica rice under Mediterranean conditions Field Crops Research 2002 83 3 251 260

[B50] Hubbard TJ Aken BL Ayling S Ballester B Beal K Bragin E Brent S Chen Y Clapham P Clarke L Ensembl 2009 Nucleic Acids Res 2009 37 Database D690 697 10.1093/nar/gkn828 19033362 PMC2686571

[B51] Liu X Wu J Wang J Zhao S Li Z Kong L Gu X Luo J Gao G WebLab: a data-centric, knowledge-sharing bioinformatic platform Nucleic Acids Res 2009 37 Web Server W33 39 10.1093/nar/gkp428 19465388 PMC2703900

[B52] Blankenberg D Taylor J Schenck I He J Zhang Y Ghent M Veeraraghavan N Albert I Miller W Makova KD A framework for collaborative analysis of ENCODE data: making large-scale analyses biologist-friendly Genome Res 2007 17 6 960 964 10.1101/gr.5578007 17568012 PMC1891355

[B53] Garris AJ Tai TH Coburn J Kresovich S McCouch S Genetic structure and diversity in Oryza sativa L Genetics 2005 169 3 1631 1638 10.1534/genetics.104.035642 15654106 PMC1449546

[B54] Sang T Ge S Genetics and phylogenetics of rice domestication Curr Opin Genet Dev 2007 17 6 533 538 10.1016/j.gde.2007.09.005 17988855

[B55] Jiao Y Wang Y Xue D Wang J Yan M Liu G Dong G Zeng D Lu Z Zhu X Regulation of OsSPL14 by OsmiR156 defines ideal plant architecture in rice Nat Genet 42 6 541 544 10.1038/ng.591 20495565

[B56] Shomura A Izawa T Ebana K Ebitani T Kanegae H Konishi S Yano M Deletion in a gene associated with grain size increased yields during rice domestication Nat Genet 2008 40 8 1023 1028 10.1038/ng.169 18604208

