# Supplementary material for: Rice-Map: a new-generation rice genome browser
Source: BMC Genomics. 2011 Mar 30;12:165. doi: 10.1186/1471-2164-12-165 (PMC3072960; doi:10.1186/1471-2164-12-165)
Supplement: Additional file 1 — Rice-Map supplementary figures. Supplementary figures about number of pre-computed tracks, inconsistent areas among different gene annotation tracks and Rice-Map user operations. [file 1471-2164-12-165-S1.PPT]

## Slide 1
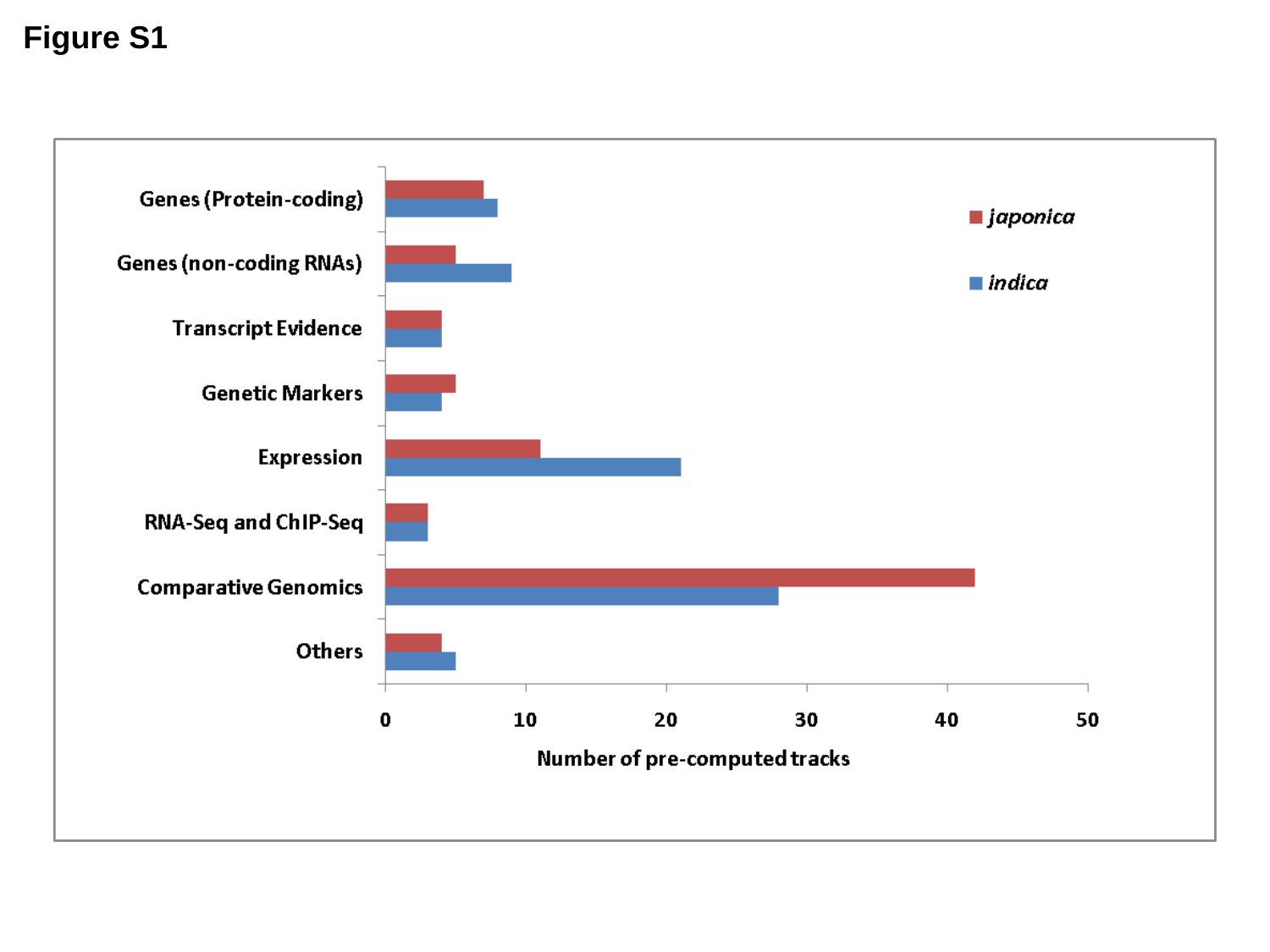

Figure S1

## Slide 2
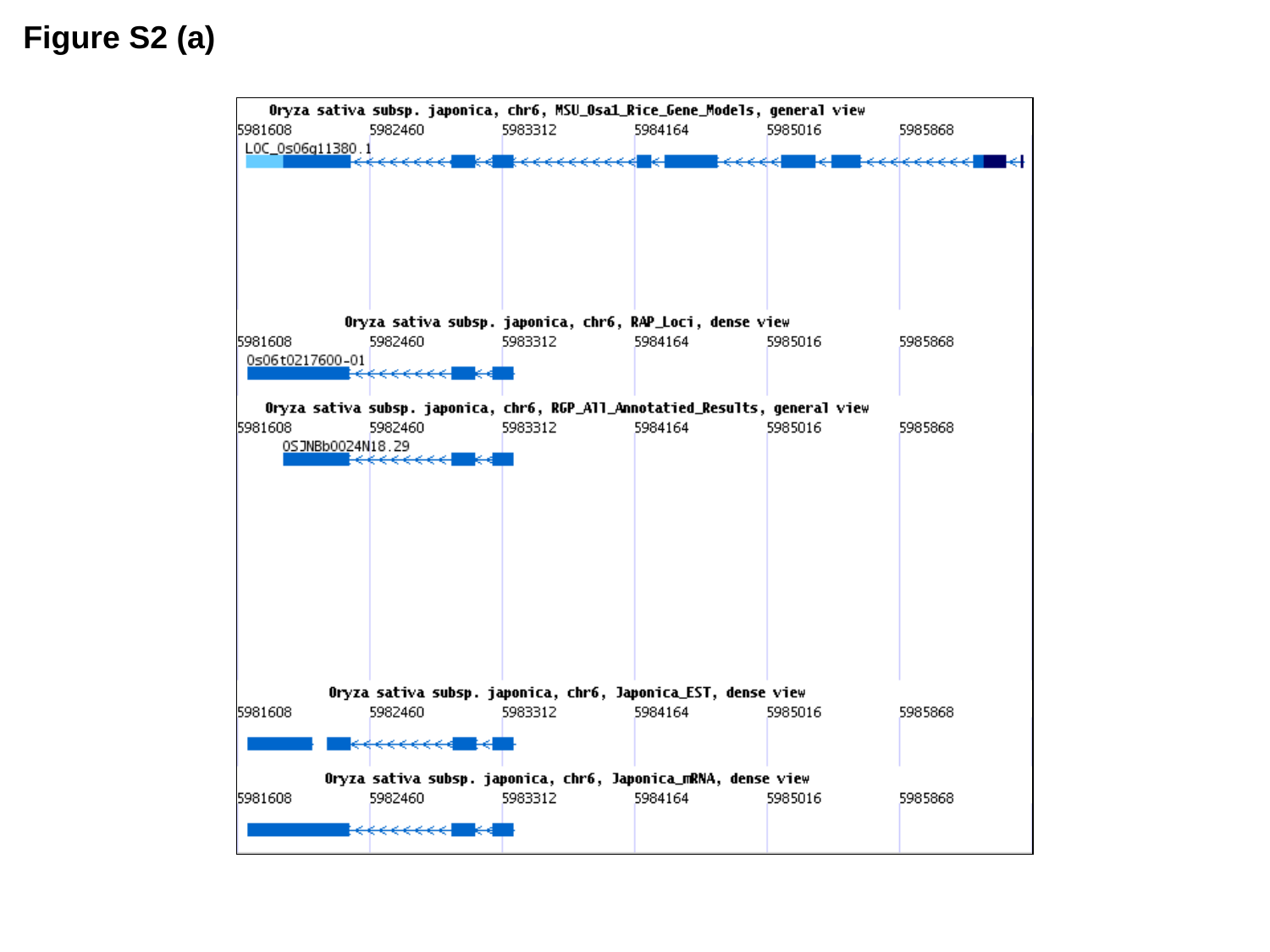

Figure S2 (a)

## Slide 3
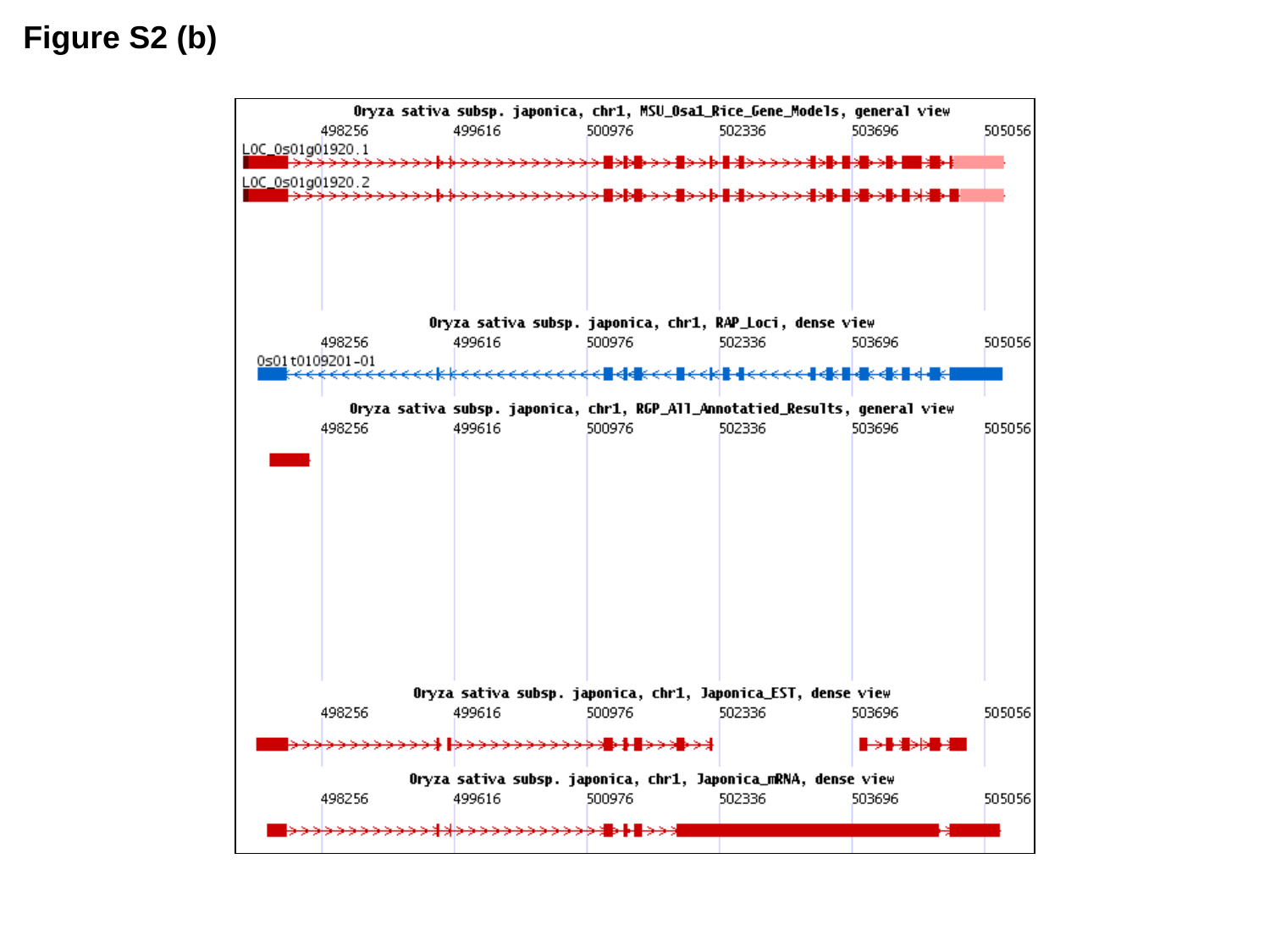

Figure S2 (b)

## Slide 4
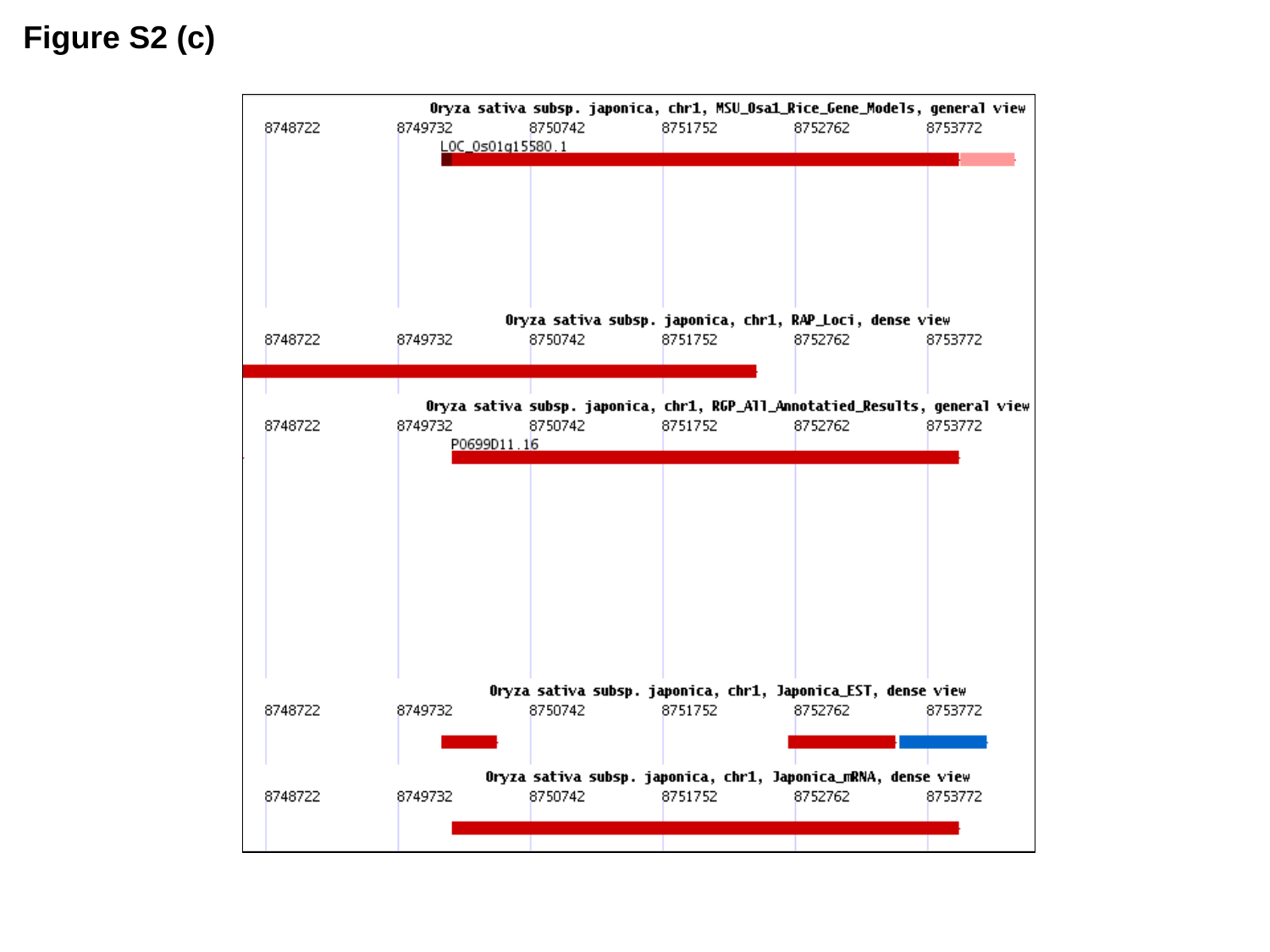

Figure S2 (c)

## Slide 5
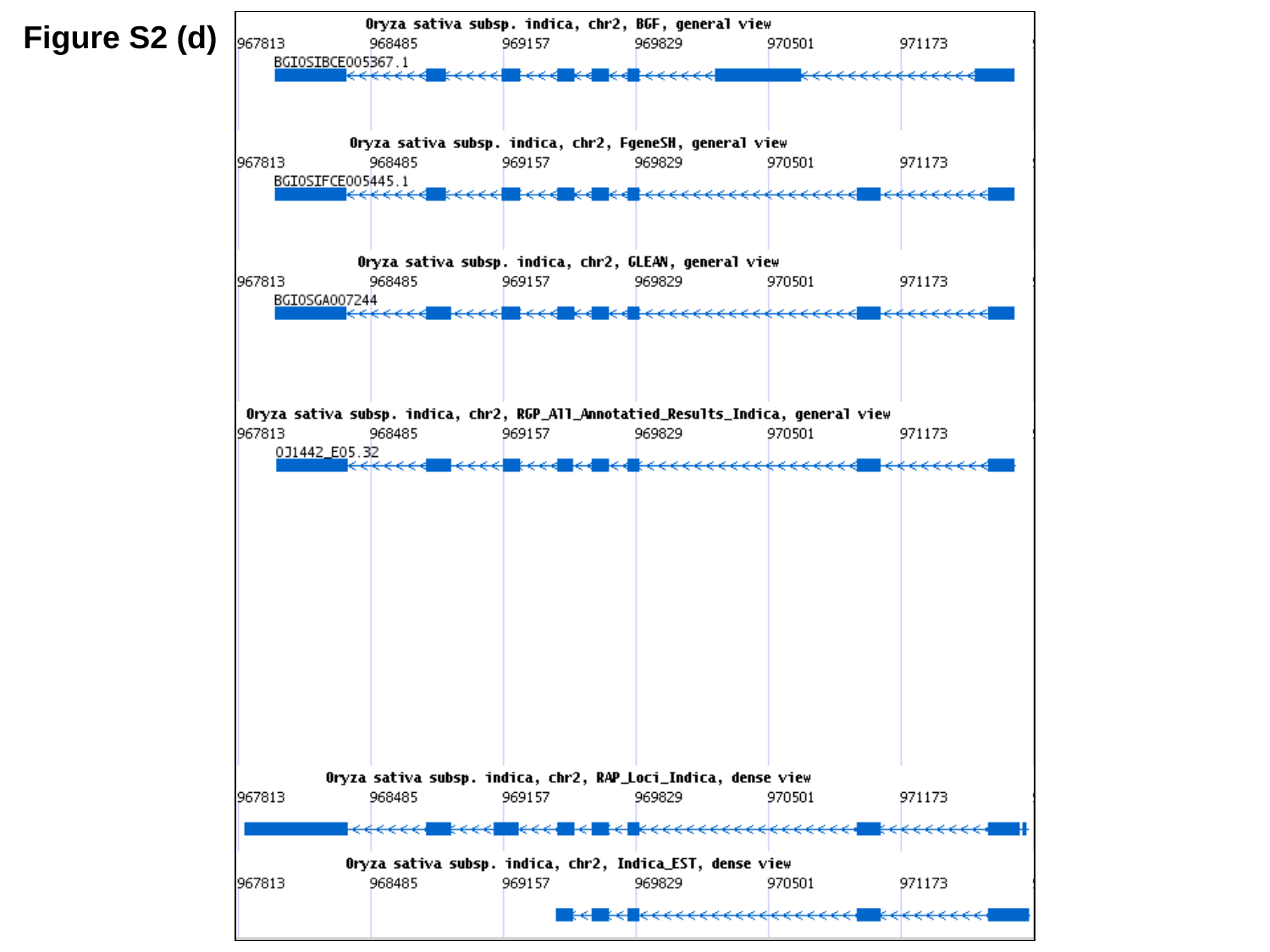

Figure S2 (d)

## Slide 6
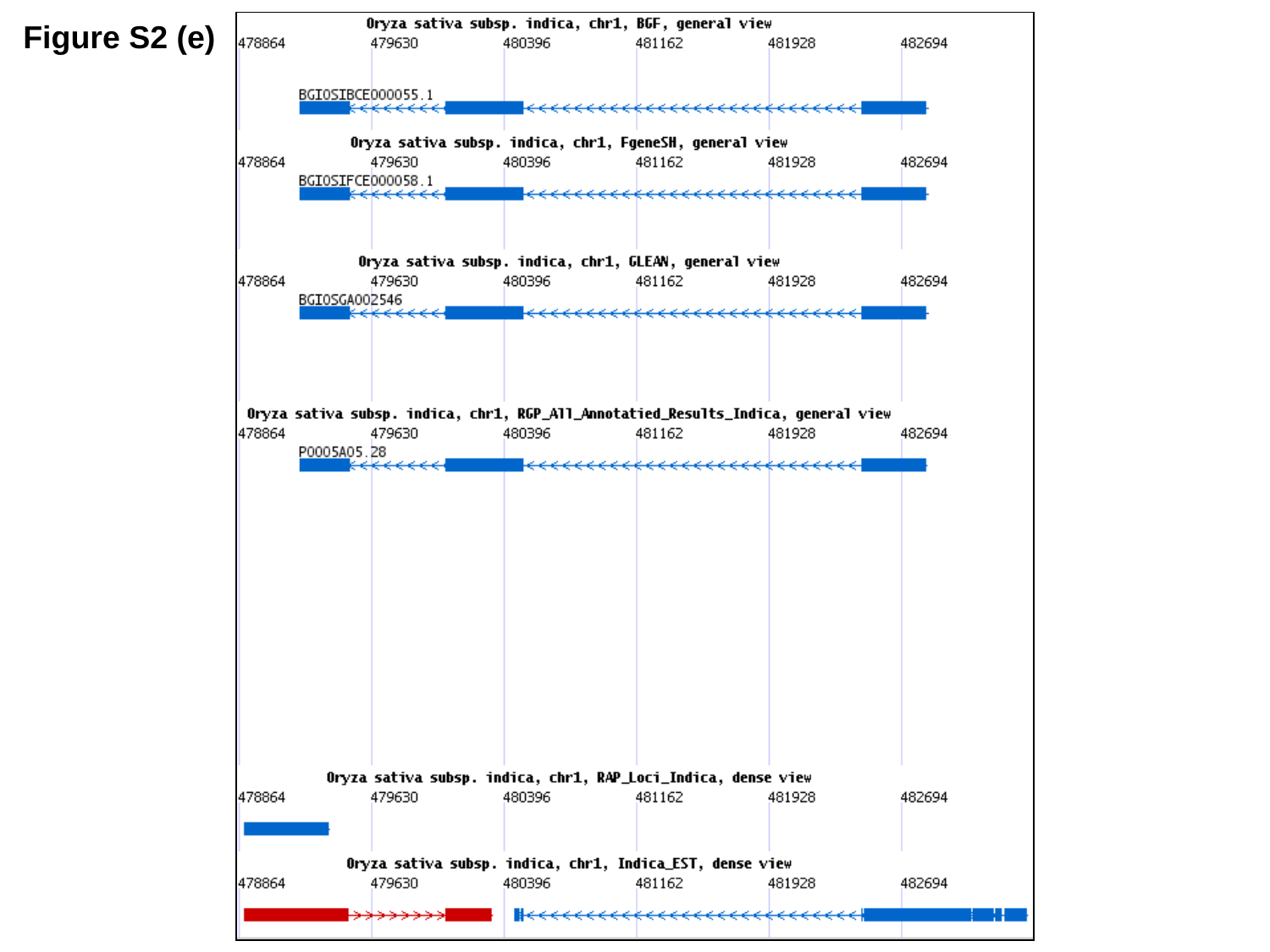

Figure S2 (e)

## Slide 7
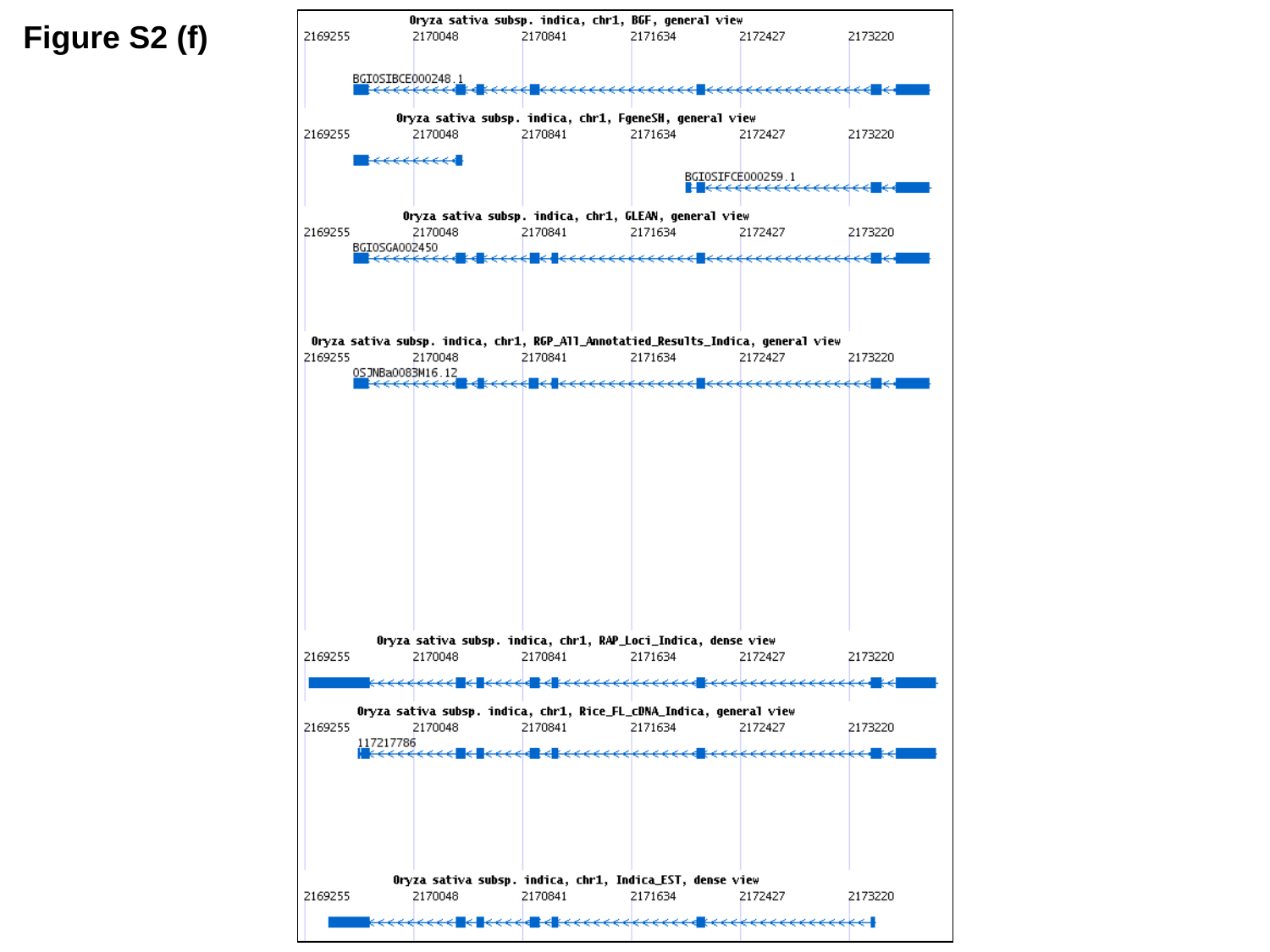

Figure S2 (f)

## Slide 8
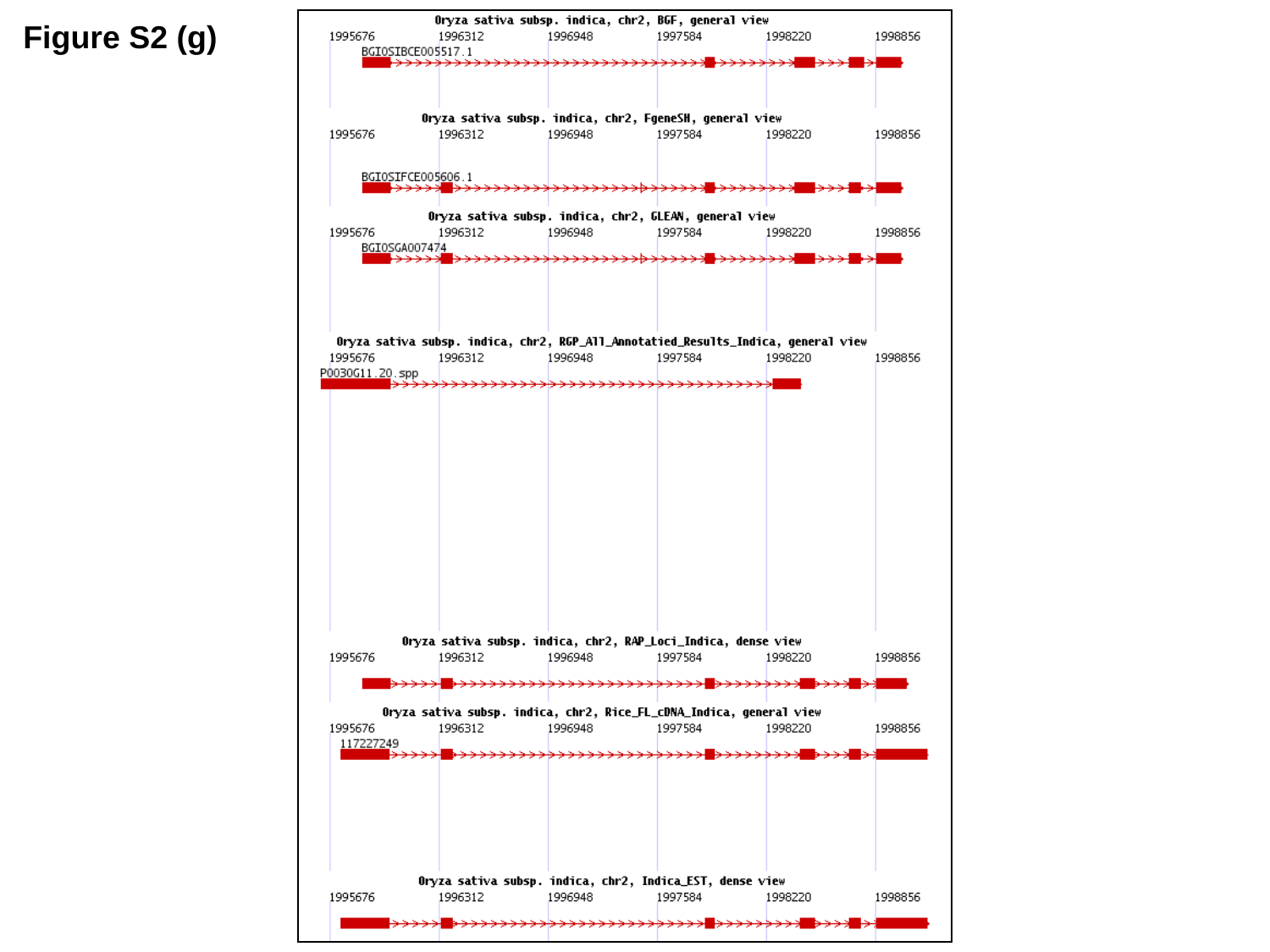

Figure S2 (g)

## Slide 9
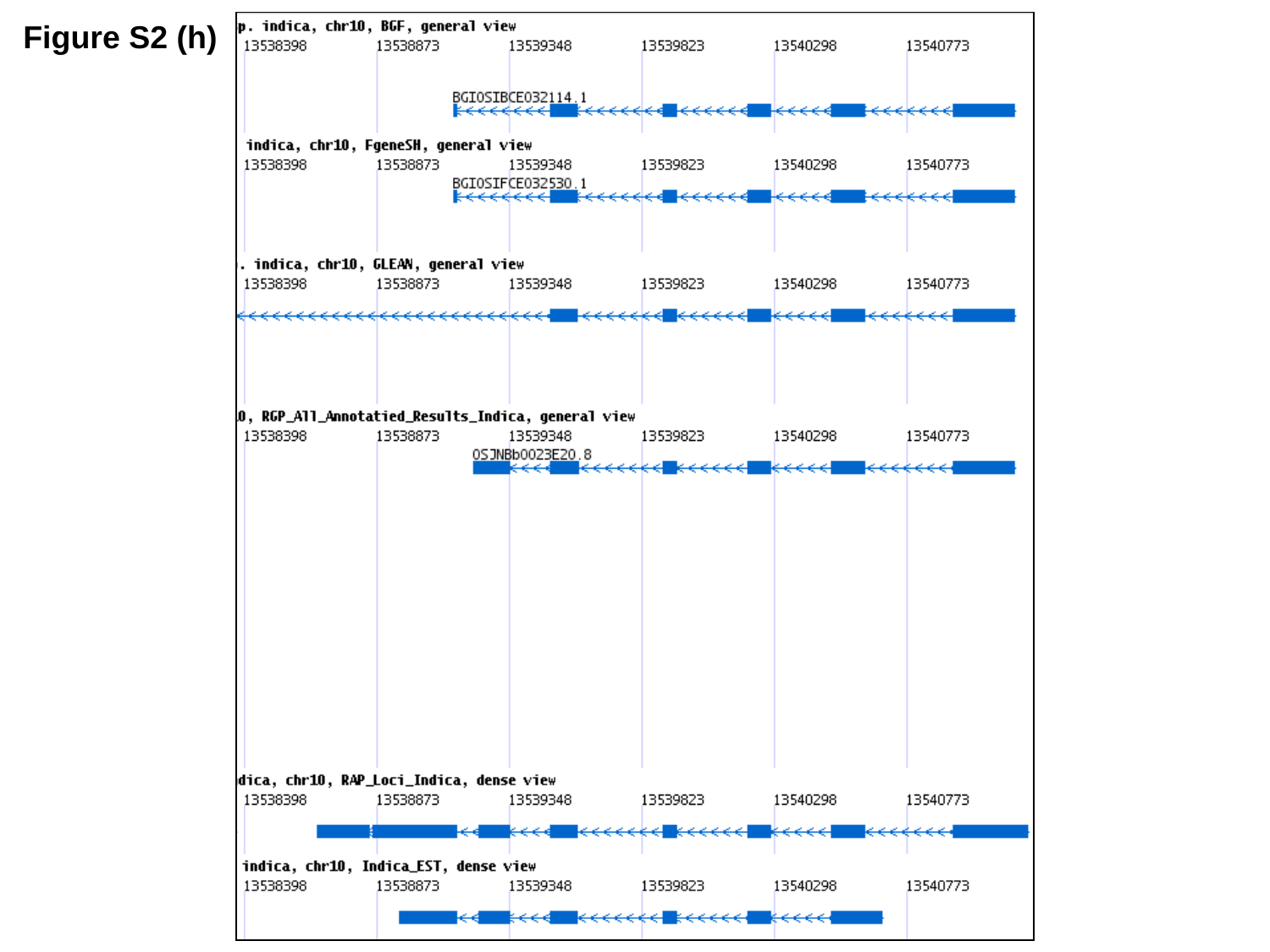

Figure S2 (h)

## Slide 10
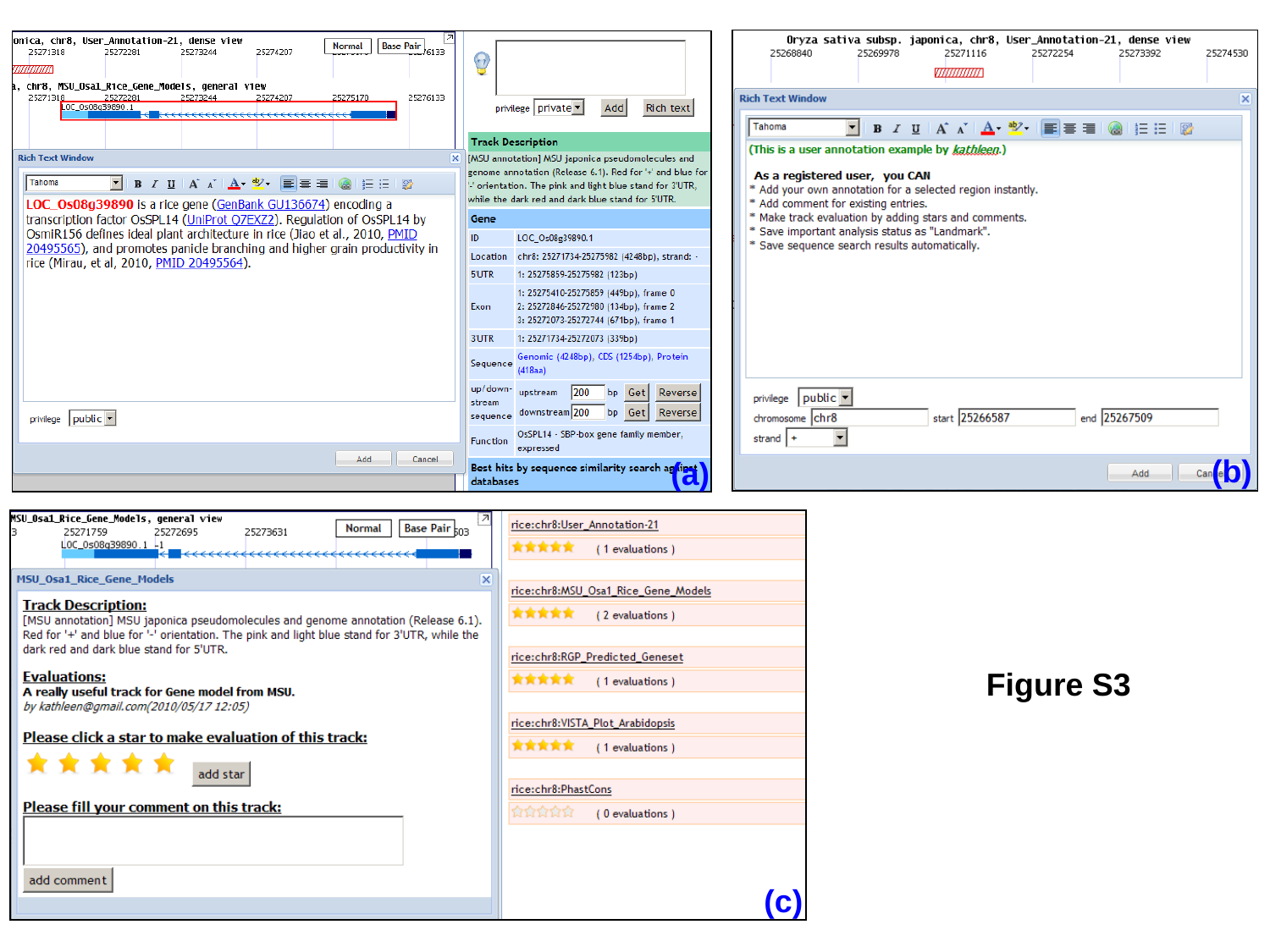

(b)
(a)
(c)
Figure S3

## Slide 11
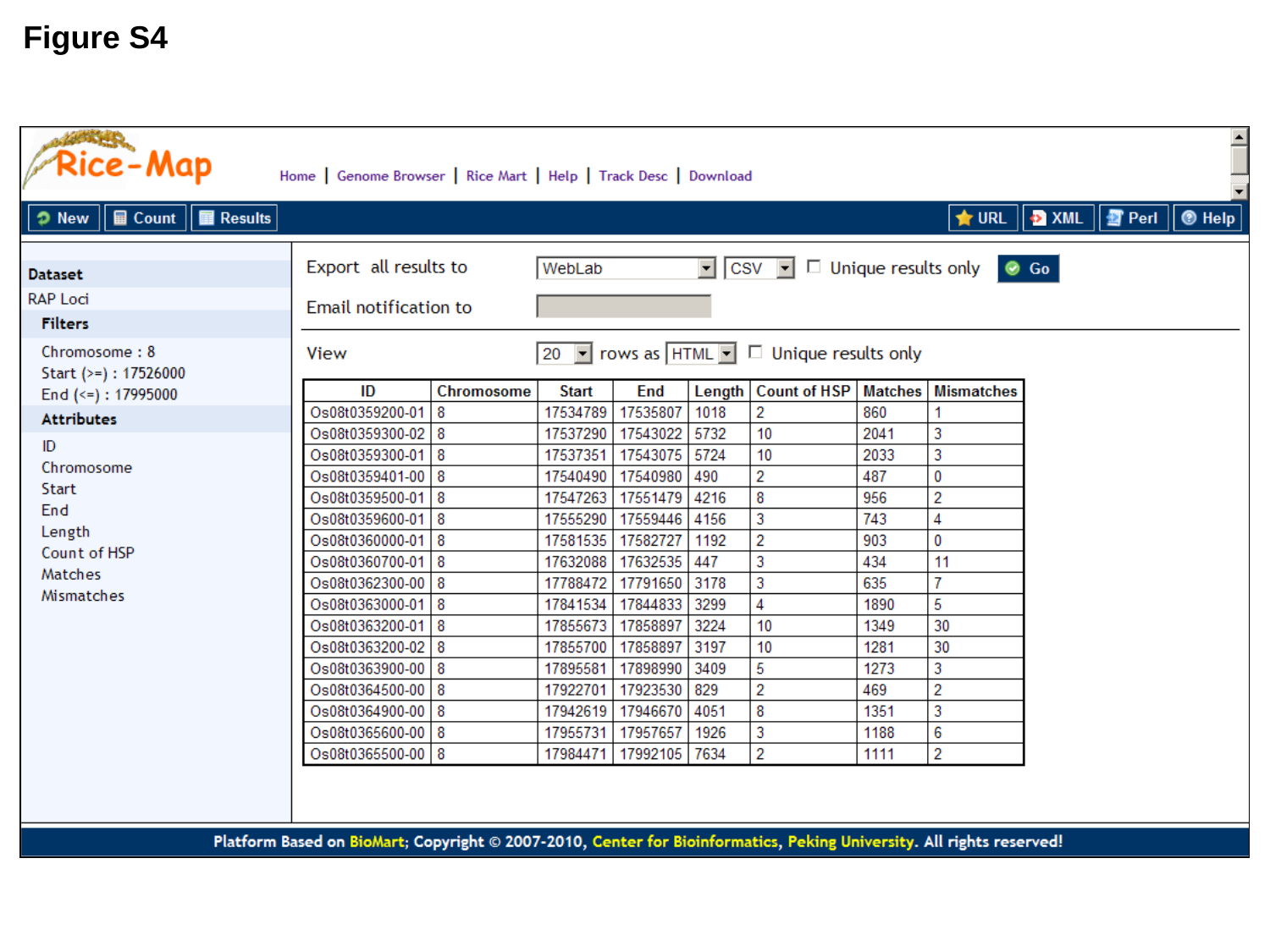

Figure S4

## Slide 12
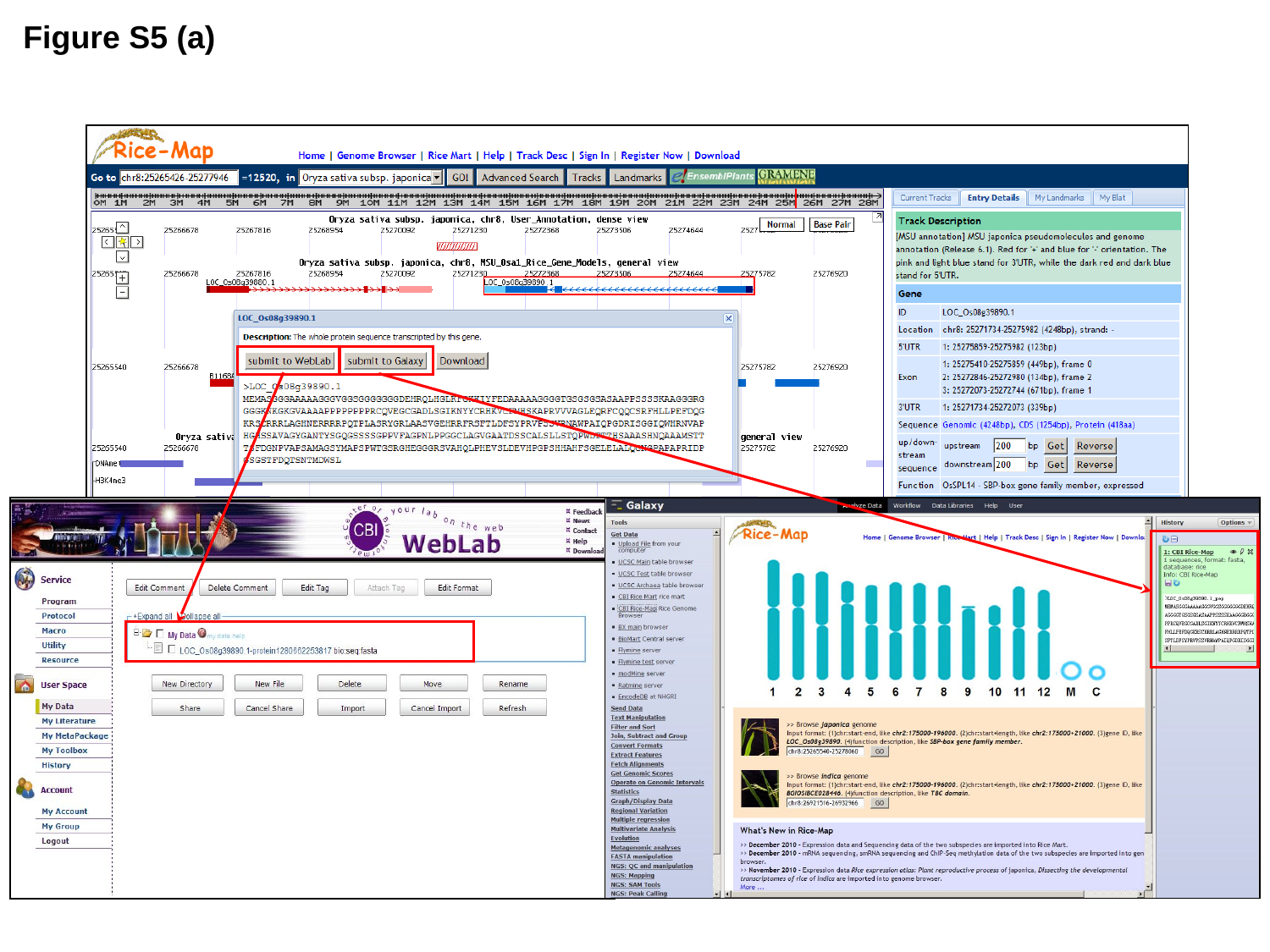

Figure S5 (a)

## Slide 13
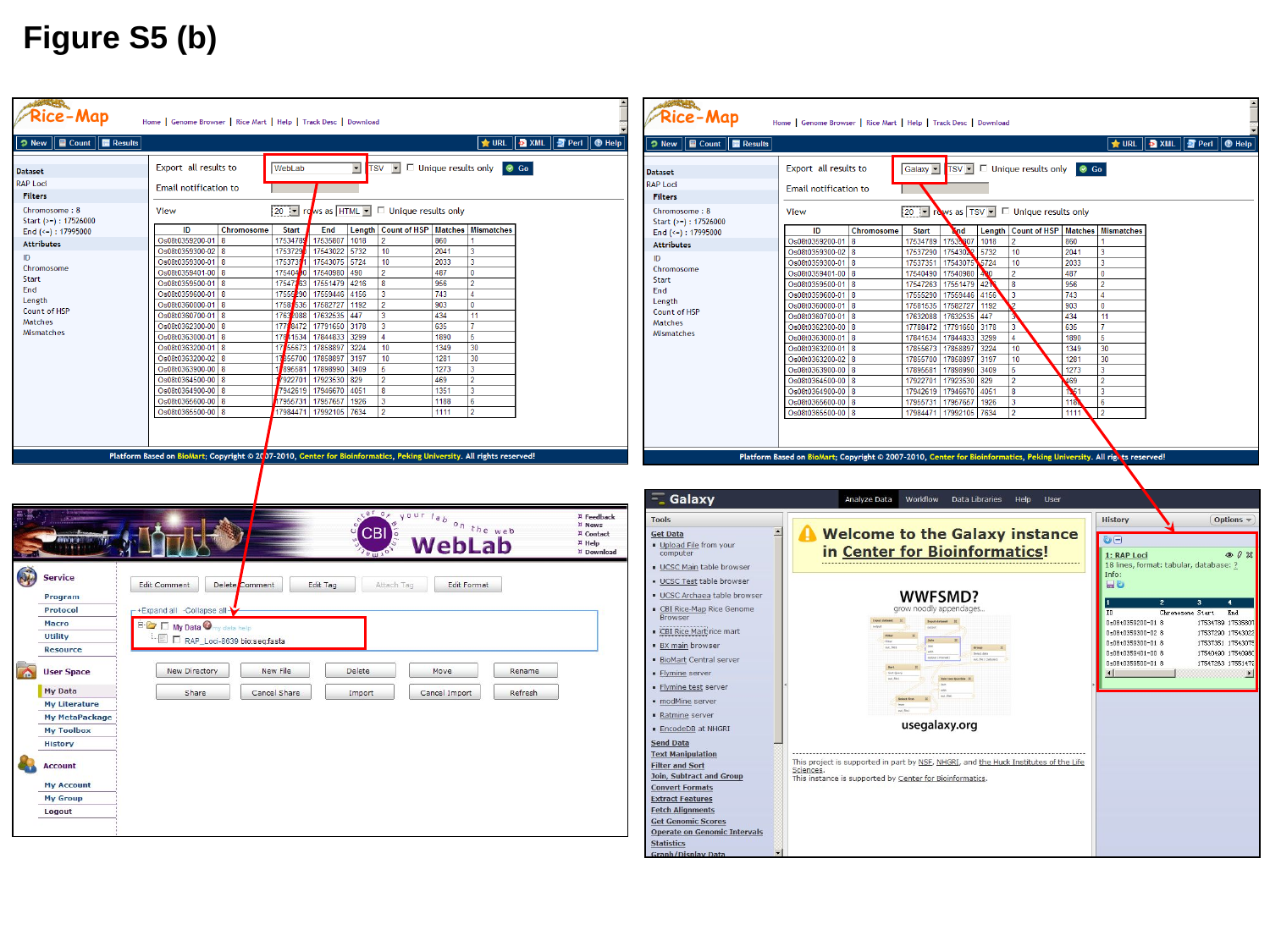

Figure S5 (b)

## Slide 14
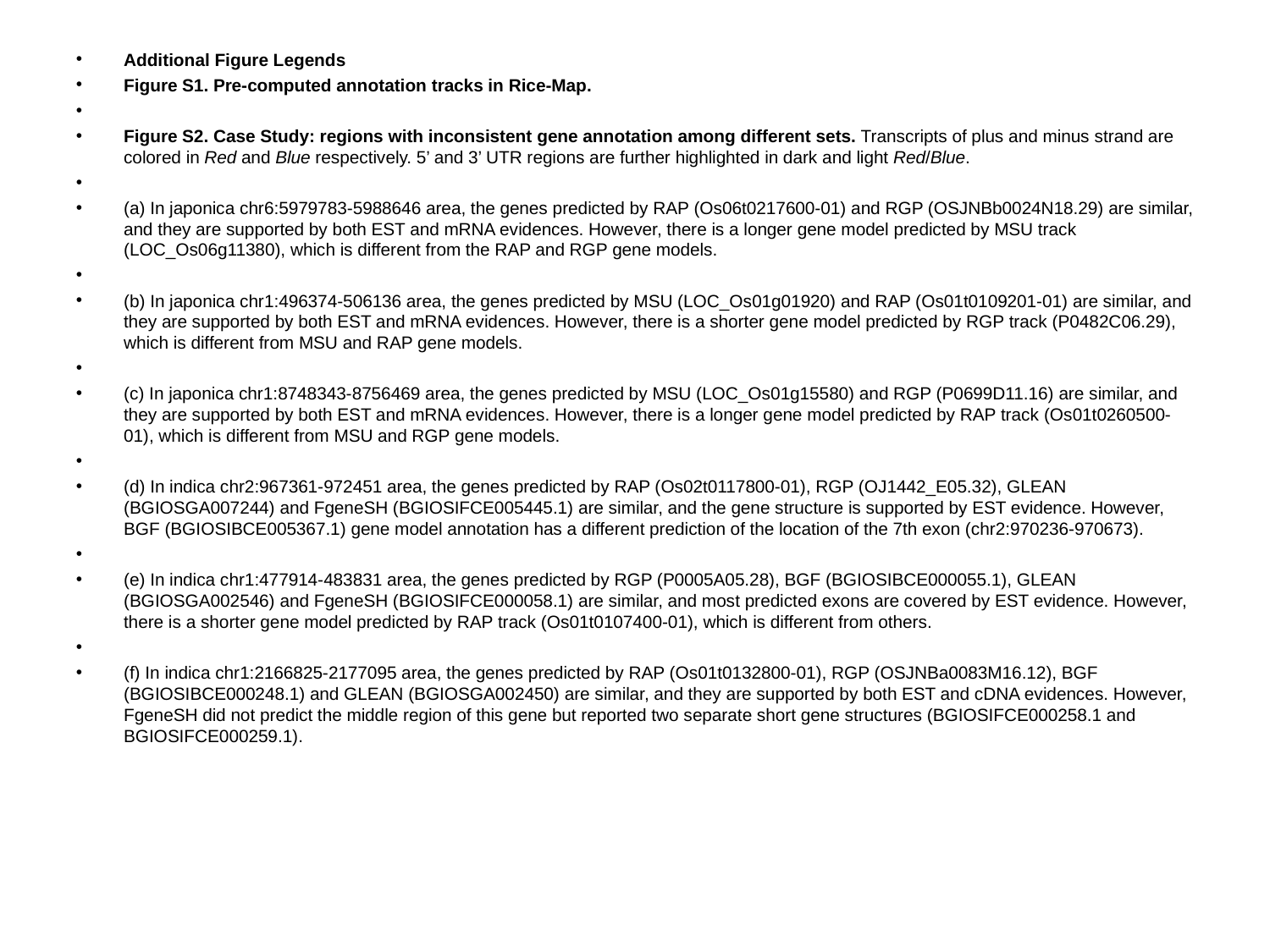

Additional Figure Legends
Figure S1. Pre-computed annotation tracks in Rice-Map.
Figure S2. Case Study: regions with inconsistent gene annotation among different sets. Transcripts of plus and minus strand are colored in Red and Blue respectively. 5’ and 3’ UTR regions are further highlighted in dark and light Red/Blue.
(a) In japonica chr6:5979783-5988646 area, the genes predicted by RAP (Os06t0217600-01) and RGP (OSJNBb0024N18.29) are similar, and they are supported by both EST and mRNA evidences. However, there is a longer gene model predicted by MSU track (LOC_Os06g11380), which is different from the RAP and RGP gene models.
(b) In japonica chr1:496374-506136 area, the genes predicted by MSU (LOC_Os01g01920) and RAP (Os01t0109201-01) are similar, and they are supported by both EST and mRNA evidences. However, there is a shorter gene model predicted by RGP track (P0482C06.29), which is different from MSU and RAP gene models.
(c) In japonica chr1:8748343-8756469 area, the genes predicted by MSU (LOC_Os01g15580) and RGP (P0699D11.16) are similar, and they are supported by both EST and mRNA evidences. However, there is a longer gene model predicted by RAP track (Os01t0260500-01), which is different from MSU and RGP gene models.
(d) In indica chr2:967361-972451 area, the genes predicted by RAP (Os02t0117800-01), RGP (OJ1442_E05.32), GLEAN (BGIOSGA007244) and FgeneSH (BGIOSIFCE005445.1) are similar, and the gene structure is supported by EST evidence. However, BGF (BGIOSIBCE005367.1) gene model annotation has a different prediction of the location of the 7th exon (chr2:970236-970673).
(e) In indica chr1:477914-483831 area, the genes predicted by RGP (P0005A05.28), BGF (BGIOSIBCE000055.1), GLEAN (BGIOSGA002546) and FgeneSH (BGIOSIFCE000058.1) are similar, and most predicted exons are covered by EST evidence. However, there is a shorter gene model predicted by RAP track (Os01t0107400-01), which is different from others.
(f) In indica chr1:2166825-2177095 area, the genes predicted by RAP (Os01t0132800-01), RGP (OSJNBa0083M16.12), BGF (BGIOSIBCE000248.1) and GLEAN (BGIOSGA002450) are similar, and they are supported by both EST and cDNA evidences. However, FgeneSH did not predict the middle region of this gene but reported two separate short gene structures (BGIOSIFCE000258.1 and BGIOSIFCE000259.1).

## Slide 15
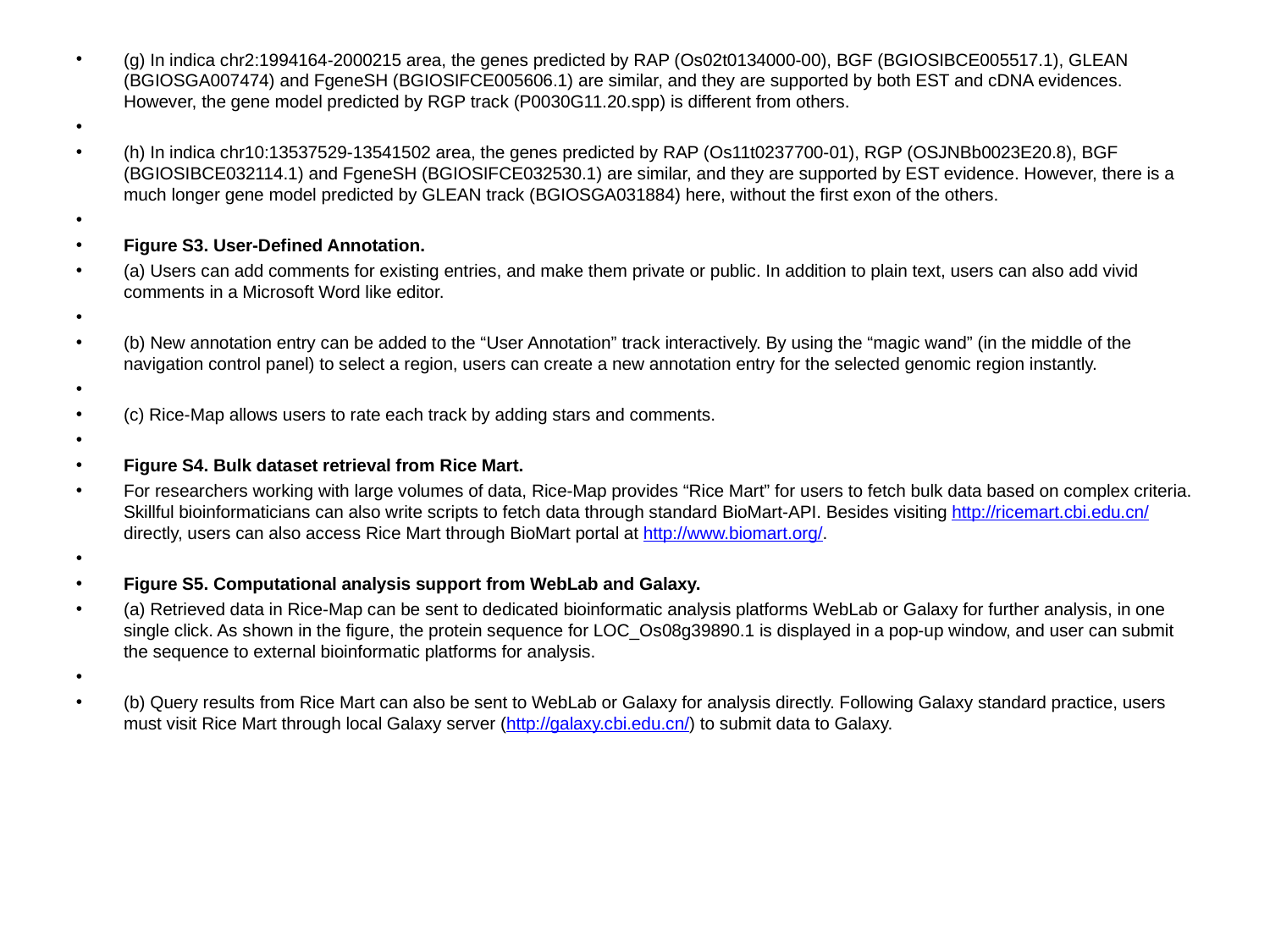

(g) In indica chr2:1994164-2000215 area, the genes predicted by RAP (Os02t0134000-00), BGF (BGIOSIBCE005517.1), GLEAN (BGIOSGA007474) and FgeneSH (BGIOSIFCE005606.1) are similar, and they are supported by both EST and cDNA evidences. However, the gene model predicted by RGP track (P0030G11.20.spp) is different from others.
(h) In indica chr10:13537529-13541502 area, the genes predicted by RAP (Os11t0237700-01), RGP (OSJNBb0023E20.8), BGF (BGIOSIBCE032114.1) and FgeneSH (BGIOSIFCE032530.1) are similar, and they are supported by EST evidence. However, there is a much longer gene model predicted by GLEAN track (BGIOSGA031884) here, without the first exon of the others.
Figure S3. User-Defined Annotation.
(a) Users can add comments for existing entries, and make them private or public. In addition to plain text, users can also add vivid comments in a Microsoft Word like editor.
(b) New annotation entry can be added to the “User Annotation” track interactively. By using the “magic wand” (in the middle of the navigation control panel) to select a region, users can create a new annotation entry for the selected genomic region instantly.
(c) Rice-Map allows users to rate each track by adding stars and comments.
Figure S4. Bulk dataset retrieval from Rice Mart.
For researchers working with large volumes of data, Rice-Map provides “Rice Mart” for users to fetch bulk data based on complex criteria. Skillful bioinformaticians can also write scripts to fetch data through standard BioMart-API. Besides visiting http://ricemart.cbi.edu.cn/ directly, users can also access Rice Mart through BioMart portal at http://www.biomart.org/.
Figure S5. Computational analysis support from WebLab and Galaxy.
(a) Retrieved data in Rice-Map can be sent to dedicated bioinformatic analysis platforms WebLab or Galaxy for further analysis, in one single click. As shown in the figure, the protein sequence for LOC_Os08g39890.1 is displayed in a pop-up window, and user can submit the sequence to external bioinformatic platforms for analysis.
(b) Query results from Rice Mart can also be sent to WebLab or Galaxy for analysis directly. Following Galaxy standard practice, users must visit Rice Mart through local Galaxy server (http://galaxy.cbi.edu.cn/) to submit data to Galaxy.
